# Endoscopic retrograde cholangiopancreatography services in Sudan during wartime: Innovations in crisis

**DOI:** 10.1055/a-2621-5666

**Published:** 2025-06-17

**Authors:** Abdelmounem Abdo, Salah Dafalla, Mohammed Bushra, Rodwan Mustafa, Reem Hamad, Ahmed Rafei, Mohamed Jaafer, Rawan Bidab, Waleed Gamus Ojan, Mohammed Ganim, Hala Abdalla

**Affiliations:** 1 535434 Gastroenterology, National Centre for Gastrointestinal and Liver Diseases, Kassala, Sudan; 2 535434 Hepatolgy, National Centre for Gastrointestinal and Liver Diseases, Khartoum, Sudan; 3 535434 National Centre for Gastrointestinal and Liver Diseases, Kassala, Sudan; 4 535434 Head of Information Technology Department, National Centre for Gastrointestinal and Liver Diseases, Kassala, Sudan; 5 535434 National Centre for Gastrointestinal and Liver Diseases, Kassala, Sudan

**Keywords:** Quality and logistical aspects, Training, Pancreatobiliary (ERCP/PTCD), ERC topics, Image and data processing, documentatiton

## Abstract

​Endoscopic retrograde cholangiopancreatography (ERCP) services in Africa have expanded in recent years, aiming to enhance healthcare infrastructure. ERCP, combining endoscopy and fluoroscopy, diagnoses and treats biliary and pancreatic ductal system conditions. Expanding these services addresses healthcare access disparities between urban and rural populations. ERCP services are well-established primarily in South Africa and Egypt. Countries like Nigeria, Uganda, Rwanda, Ethiopia, and Kenya have sustained ERCP services over the past 5 years. This paper examines the need to expand ERCP services as a step toward health equity in Africa. In Sudan, ERCP services began in 1982 at Soba University Hospital, followed by Ibnsina Specialized Hospital. Before the conflict, only seven centers in Khartoum offered ERCP services. The war that started in April 2023 severely disrupted healthcare services, including ERCP. The National Center for Gastrointestinal and Liver Diseases relocated to Madani City in June 2023, resuming ERCP services by July. By December 2023, 375 procedures were performed. Following further conflict, services moved to Kassala City, where 420 ERCP cases were completed between March and December 2024.​ Innovative solutions addressed challenges such as equipment compatibility and accessory availability. A fluoroscopy machine was adapted from a non-functional urology lithotripsy system, and locally designed tables improved procedural efficiency. Development of a local scoring system for trainee assessment after 50 supervised procedures aims to establish a sustainable ERCP fellowship program, addressing the shortage of specialists. These efforts highlight resilience and innovation in delivering ERCP services during conflict.

## Introduction

In recent years, expansion of endoscopic retrograde cholangiopancreatography (ERCP) services has emerged as a focal point in ongoing efforts to enhance healthcare infrastructure in Africa. ERCP, a sophisticated technique combining endoscopy and fluoroscopy to diagnose and treat conditions of the biliary or pancreatic ductal systems, holds significant promise for the continent. However, broader implications of expanding ERCP services extend beyond immediate medical benefits. Introduction and proliferation of such advanced diagnostic and therapeutic procedures addresses the stark disparities in healthcare access between urban and rural populations. By bringing state-of-the-art medical technology to underserved areas, Africa stands on the cusp of fostering a more equitable healthcare landscape.

ERCP services in Africa are not well established or sustained, except in South Africa, and Egypt 1 2 . However, a few other African countries, including Nigeria, Uganda, Rwanda, Ethiopia, and Kenya, have managed to sustain ERCP services over the past 5 years 3 4 5 .

This paper, therefore, examines the urgent need to expand ERCP services, not only as a medical necessity but also as a fundamental step toward social justice, bridging the healthcare divide and promoting greater health equity across Africa.

## Sustainability of ERCP services in Africa faces several challenges

First, training and retention of personnel remain significant obstacles. ERCP requires well-trained professionals, and achieving competency necessitates performing a certain number of supervised procedures. However, retaining skilled staff in many African countries is difficult due to limited career incentives, workforce migration, and resource constraints.

Second, the high cost and limited accessibility of accessories hinder service expansion. Last, lack of maintenance and repair services for fluoroscopy and endoscopy equipment leads to frequent downtime, disrupting service continuity and limiting patient access.

The vicious cycle of ERCP equipment and personnel shortages further exacerbates these challenges, potentially leading to the collapse of ERCP programs in some regions.

## ERCP services in Sudan

ERCP services began in Sudan in 1982 at Soba University Hospital. Followed by Ibnsina Specialized Hospital 6 . Until the onset of the war, ERCP services were unavailable outside of Khartoum (Only seven centers, three governmental and four private). To further understand the complexity of ERCP services in Sudan, it is essential to analyze how medical advancements and socioeconomic barriers have influenced their implementation. The inception of ERCP procedures in Sudanese hospitals, particularly in Khartoum, marked a significant leap forward for gastrointestinal diagnostics. Despite this progress, the socioeconomic landscape created substantial hurdles. Rural areas continued to suffer from a stark lack of medical infrastructure and trained professionals, limiting the reach of these advanced diagnostic tools. The urban-rural divide in access to ERCP services has thus magnified existing health inequities. Data from three hospitals in Khartoum revealed that while urban centers were capable of executing complex procedures and measuring radiation doses effectively during ERCP, similar facilities were often nonexistent in rural regions 7 . These disparities are not merely logistical but also socioeconomic; patients from marginalized communities face financial constraints that impede their ability to seek advanced medical care even when available. This highlights the disconnect between medical progress and socioeconomic challenges in Sudan's healthcare system.

In mid-April 2023, Sudan's already fragile healthcare system came under increasing strain as the conflict between the Rapid Support Forces and the Sudanese Armed Forces spread beyond Khartoum into neighboring states, further exacerbating pre-existing epidemiological challenges 8 . The situation deteriorated rapidly, with 30.4 million of the country’s 50.04 million population in need of humanitarian assistance 9 . The humanitarian crisis deepened in 2024 when a large-scale cholera outbreak placed an overwhelming burden on the healthcare system 10 .

Following the outbreak of war on April 15, 2023, the number of displaced individuals surged, with more than half of the Sudanese population displaced. All health services—including advanced procedures such as ERCP—collapsed nationwide 10 . In response, the National Center for Gastrointestinal and Liver Diseases successfully relocated to Madani City in June 2023 and began efforts to re-establish ERCP services. By the end of July, essential equipment including an endoscopic scope, C-arm, and accessories had been procured, allowing ERCP services to resume. During this period, two junior gastroenterologists and a team of endoscopy nurses were trained. Each trainee had access to supervised training, handling up to 40 cases per month. By December 15, 2023, we had performed 375 ERCPs 11 .

Unfortunately, on December 15, 2023, the war extended to Madani City, causing another collapse of health services in the region. It took us another 3 months to relocate, this time to the eastern part of the country, to Kassala City.

From March 26 to December 31, Kassala Center performed 420 ERCP cases and 83% of patients travelled from outside Kassala state. Across three weekly lists, each list included a trainee who had completed gastroenterology and hepatology training.

## Pioneering solution to overcome the obstacles: Learning Lessons from war

**Fig. 1:**
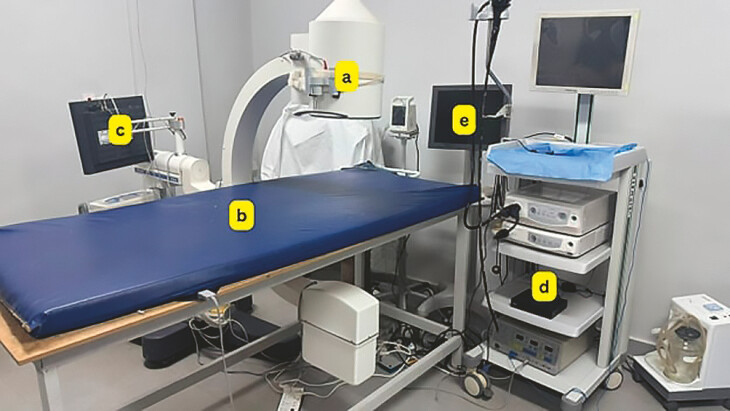
Overcoming fluoroscopy-related challenges in ERCP procedures at Kassala Diagnostic Center. **a** The repurposed C-arm fluoroscopy machine from a non-functioning urology lithotripsy unit. **b** A locally designed special procedure table to improve compatibility with the fluoroscopy system for ERCP. **c** An innovative solution for image mirroring issues by fixing the fluoroscopy screen upside down for correct orientation during procedures. **d** Implementation of DVR technology, originally designed for security cameras, repurposed for fluoroscopic imaging. **e** Integration of DVR technology with an external display screen to enhance real-time visualization and data sharing during ERCP procedures.

**Fig. 2:**
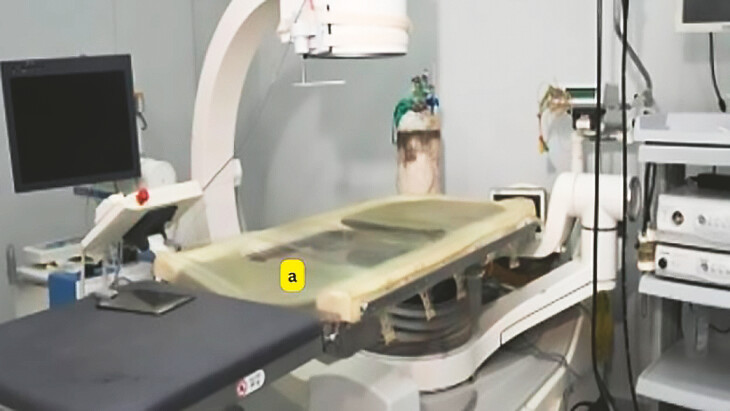
The original urology procedure table, which posed maneuverability challenges for the C-arm during ERCP procedures.

One significant challenge in Kassala Medical Center was acquisition and effective use of a fluoroscopy machine ( Fig. 1 **a** ), necessary for various imaging procedures. Initially, the center repurposed a C-arm from a non-functioning urology lithotripsy machine; although the lithotripsy component was defunct, the C-arm remained operational and capable of performing fluoroscopic imaging. The primary obstacle that arose involved the compatibility of this equipment with the requirement for the ERCP procedure, particularly concerning the procedure table. The table had been customized specifically for urology services ( Fig. 2 **a** ), which introduced difficulties when attempting to maneuver the C-arm around it. This specialized design meant that staff often struggled to position the C-arm appropriately, hindering smooth operation during procedures. Addressing this mismatch required creative adaptations and potentially increased procedure times as personnel navigated these spatial constraints. A special table ( Fig. 1 **b** ) was designed locally under the supervision of the medical staff.

The mirroring phenomenon in fluoroscopic imaging presents a unique complexity, particularly observable in procedures such as ERCP. Specifically, the fluoroscopy image of lithotripsy C-arm which was found at Kassala Diagnostic Center inherently involves right and left mirroring but does not exhibit an up-down reversal. This characteristic is crucial for accurate interpretation during medical procedures. To address the dual challenges associated with the display of fluoroscopic images during procedures, a locally designed table and adjustable screen system were implemented. This innovative approach resolved initial issues effectively by fixing the screen upside down ( Fig. 1 **c** ).

However, after completing the first five cases, a new obstacle emerged: difficulty displaying fluoroscopic images on a single fixed screen attached to the C-arm ( Fig. 1 **c** ), which needed to be simultaneously visible to both professional groups. The staff ingeniously tried to manage this challenge by integrating additional screens or repositioning existing monitors so as to allow real-time visual access for both radiologists and endoscopists, but this solution failed. Implementing digital video recording (DVR) ( Fig. 1 **d** ) technology originally intended for security cameras has proven to be an innovative solution in the medical field, particularly in integration of C-arm imaging systems with external display screens ( Fig. 1 **e** ). By connecting the C-arm to another screen through DVR technology, we have effectively resolved issues related to real-time visualization and data-sharing during procedures. This system is not only useful for providing immediate access to fluoroscopic and endoscopic views but also offers the capacity to record these cases comprehensively. The ability to archive procedure footage ensures that detailed reviews can be conducted postoperatively, which enhances both learning and quality assurance processes. In addition, there is considerable potential for this technology in educational and collaborative settings. The center is currently evaluating its application for live demonstrations and conference participation, a domain for which it has previously relied on professional teams for execution. Transitioning this capability in-house by leveraging existing DVR technologies will streamline operations and potentially reduce costs while maintaining the high standards of visual fidelity necessary for effective communication within the medical community.

## Facing numerous challenges in development of medical accessories

Our team has achieved significant milestones by developing innovative solutions tailored to meet specific clinical needs, including adaptation and reimbursement of unavailable accessories such as the precut knife and 7F pusher. One noteworthy achievement is creation of precut devices from the sphincterotome and polypectomy snare, which enhance precision and reduce procedure times during endoscopic intervention. Furthermore, we have engineered a 7F pusher from an injector needle, streamlining stent deployment and improving patient outcomes through minimally invasive techniques ( **Supplementary Video 1** ). These advancements not only address previously encountered obstacles but also establish new standards in medical accessory design, underscoring our commitment to enhancing procedure efficiency and patient safety.

The benefits of ERCP are extensive, because its ability to diagnose and treat complex conditions of the biliary and pancreatic conditions significantly reduces morbidity and mortality rates. In addition, expanding these services requires investment in specialized training for healthcare professionals, which in turn builds local expertise and capacity. This not only raises the standard of care but also empowers medical practitioners within their communities. Furthermore, equitable distribution of advanced medical technologies fosters an inclusive healthcare system, ensuring that no individual is deprived of critical health interventions due to their geographical or socioeconomic status 12 .

## ERCP fellowship in Sudan

Given the high volume of ERCP cases we encounter, we are considering establishing an ERCP fellowship program over 1 year. A significant challenge in such a program would be assessing trainee progress and skills. Initially, we considered using the Diagnostic and Procedural Outcomes Scale developed by the Joint Advisory Group (JAG). However, we found it excessively detailed and lengthy for our context. As a result, we are contemplating developing a local scoring system that can be completed electronically by trainees. We are developing a streamlined, electronic scoring system that allows for efficient conscious assessment of trainee competency. This local scoring system aims to build a robust, sustainable ERCP training pipeline to support Sudan’s healthcare needs despite current challenges. The system would be implemented after each trainee has completed at least 50 procedures. Key criteria and scoring points are shown in ( Table 1 ).

**Table:** **Table 1** Sudan training assessment of ERCP: Abdo State.

Criterion	Description	Points
Case Complexity	Highly complex	3 points
	Moderately complex	2 points
	Not complex	1 point
Requirement for supervisor assistance	No assistance required	3 points
	Assistance required	0 points
Type of assistance required	Precut	2 points
	Sphincterotomy	1 point
	Extraction	1 point
	Stenting	1 point
	Cannulation	0 points
	Supervisor failed to assist	3 points

Trainees will be assessed using this scoring system after completing 50 supervised procedures, with the goal of evaluating their competency after reaching 100 supervised procedures. The scoring system was validated through assessment of four trainees at our center. Each had completed 100 ERCP procedures—50 prior to and 50 following implementation of the scoring system. Three of these trainees were deemed competent to practice independently.

This structured scoring system is designed to ensure that trainees develop the necessary technical skills and decision-making abilities for safe, independent ERCP practice, given the shortage of ERCP specialists in Sudan.

Each procedure has a maximum score of six points, based on case complexity, independence, and type of assistance required. After 50 procedures, the evaluation focuses on key performance indicators (KPIs). The primary KPI is successful cannulation without assistance, which awards the trainee the maximum of three points. If cannulation is unsuccessful, the trainee receives the lowest score; however, a positive score may still be assigned depending on the type and level of assistance required.

Notably, a trainee may receive the maximum score if the trainer was unable to perform successful cannulation after the trainee’s attempt. The trainee’s cumulative score will then be used to assess their readiness for independent practice ( Table 2 ). Taking this into consideration, ensuring that independent trainees consistently meet competency standards is essential. A follow-up reevaluation 6 to 12 months after beginning independent practice will help maintain skills and quality standards, especially for sole ERCP specialists in a region. Conducting an intermediate evaluation after 20 to 30 procedures could provide valuable feedback early, allowing trainees to address specific areas for improvement sooner. This approach prevents procedure errors from accumulating and strengthens performance by the time they reach 50 procedures.

**Table:** **Table 2** Abdo State scoring thresholds and outcomes for ERCP fellowship competency.

Score range	Outcome	Next steps
250+ points	Independent practice granted	Competency confirmation after 6–12 months of independent practice
200–250 points	Targeted training and reevaluation	Additional training focused on identified weak areas, followed by reevaluation
Below 200 points	Extended training and periodic assessment	Continued supervised training with periodic assessments for progress and readiness for reevaluation

This scoring system will undergo further validation. Its successful implementation is expected to significantly address the shortage of ERCP service providers in Sudan. International guidelines, such as those from the Conjoint Committee for the Recognition of Training and the American Society for Gastrointestinal Endoscopy (ASGE)—which recommend a minimum of 200 unassisted procedures 13 14 —and the JAG, which recommends 300 procedures 15 , emphasize the importance of procedure volume in ensuring quality and competency in endoscopic practice.

However, our recent experience—conducting over 900 ERCP procedures within 18 months at a single center during wartime, with only one trainer and four trainees—demonstrates the urgent need for a localized, scalable approach. This experience led to development of this scoring system as a means to decentralize this highly specialized service, expand training of gastroenterologists, and maintain service quality and patient safety. Ultimately, this initiative aims to establish a well-structured, dedicated ERCP fellowship program in Sudan.

## Conclusions

Ultimately, prioritizing expansion of ERCP services symbolizes a commitment to achieving better health equity, ensuring that all populations benefit from advancements in medical science and technology. Expansion of ERCP services in Africa represents more than a leap forward in medical capability; it is a crucial step toward addressing healthcare inequities across the continent, by integrating advanced diagnostic and therapeutic technologies into both urban and rural settings. Proliferation of ERCP services is a hallmark of progress that underscores our commitment to fostering health equity throughout Sudan. As we move forward, let this initiative inspire further advancements that continue to narrow the healthcare divide, ensuring better access and outcomes for all communities.
